# Assessing the United Nations sustainable development goals from the inclusive wealth perspective

**DOI:** 10.1038/s41598-023-28540-0

**Published:** 2023-01-28

**Authors:** Yogi Sugiawan, Robi Kurniawan, Shunsuke Managi

**Affiliations:** 1 National Research and Innovation Agency of Indonesia (BRIN), Gedung 90 , Kawasan Puspiptek Serpong, South Tangerang, 15340 Indonesia; 2 Ministry of Energy and Mineral Resources, Jalan Pegangsaan Timur No. 1a, Jakarta, 10320 Indonesia; 3 Urban Institute, Kyushu University, 744 Motooka, Nishi-ku, Fukuoka, 819-0395 Japan

**Keywords:** Sustainability, Socioeconomic scenarios

## Abstract

The statement of sustainability in the sustainable development goals (SDGs) framework needs to be supplemented by a formal proof that intergenerational well-being also improves. This is the first study that aims to provide empirical evidence that links the progress of the SDGs and the changes in well-being, which are proxied by the SDG Index and the Inclusive Wealth (IW) Index, respectively. We propose an SDGs-wealth model which was analyzed using a machine learning method involving a balanced panel of 147 countries for 2000–2019. We find a strong correlation between wealth and the SDGs, with Goals 12, 13, and 7 being the most significant predictors of wealth. In contrast to Goals 12 and 13, we find a positive correlation between Goal 7 and the per capita IW Index, suggesting that promoting affordable and clean energy is beneficial for wealth accumulation. Quite the opposite, fostering responsible consumption and production and climate actions might be detrimental to wealth. We also find an alarming result for 50 countries in our study since they have deviated from the sustainable development trajectories either in the short or long run. Our study suggests that to achieve sustainable development, instead of focusing on the complex interactions among the SDGs, policymakers should put a stronger focus on improving IW.

## Introduction

Excessive reliance on the gross domestic product (GDP) as the main indicator for measuring welfare has led to various problems that threaten the sustainability of economic development, such as increasing income inequality and rapid depletion of natural resources. GDP is undeniably a good measure of marketed economic activity. However, it is inadequate and should not be used for measuring welfare because there exist numerous non-marketable assets that matter to welfare but are not considered in GDP^1,2^. As a result, development policies that focus only on pursuing GDP growth tend to deviate from the sustainable development path, which eventually leads to a potential conflict between maintaining the GDP growth, for the sake of the current generation, or preserving nature, for the sake of the future generations^3^. Development that sustains needs to consider not only socioeconomic but also environmental aspects so that the well-being of future generations will not be declined^4,5^.

Societies and policymakers alike are keenly interested in whether the national economy’s achievement, as measured by GDP growth, follows the path of sustainable development. The sustainable development goals (SDGs), which were adopted in 2015 by the United Nations General Assembly as a part of the 2030 Agenda for Sustainable Development, provide guidelines for a sustainable development path aiming to balance the development of three main pillars, i.e., economy, society, and environment. Compared to the conventional development framework, the SDGs provide a comprehensive and multidimensional approach^6^. Hence, instead of solely relying on GDP growth, the progress toward sustainable development in the SDG framework is measured by 17 goals, which are further specified into 169 targets and 247 indicators. In order to formulate forward-looking policy measures, it is necessary to look backward, through monitoring and evaluation^7^. For this purpose, various indicators that portray progress in both socioeconomic and environmental aspects are required. However, at the global level, there are only 68% of environmental-related indicators are widely available^8^.

To track global efforts toward sustainable development and guide policy development and implementation, it is crucial to quantify the progress toward achieving the SDGs. Having done so, the world needs to monitor progress towards the SDGs by assessing historical and current conditions at global, national, and different regional levels^9^. As it represents the considerable and crucial change that our society is implementing, it also requires a large number of actors^7^. Measuring sustainable development is often complex, interdependent, and hard to comprehend, indicating that it requires a holistic perspective.

A composite index or series of indicators has been utilized to measure the progress toward the SDGs^10^. On the one hand, using a series of indicators that cover rich data allows an examination of unbalanced progress between targets and their potential interactions^11^. Therefore, utilizing a large number of sub-indicators creates a difficult interpretation^12^. Involving tens or hundreds of sub-indicators may also increase user's burden in selecting appropriate indicators^13^. On the other hand, composite indices provide a clear snapshot that can attract attention, showing a summary of overall progress as well as serving international and cross-country comparisons^14^. Even so, a single index might conceal the relationship between goals and the potential imbalance between the three pillars: economy, society, and environment^10,15^. It might also collapse complexity into composite scores^13^.

The 17 goals of the SDGs are a set of indivisible goals which form a network of targets that are universally applicable, irrespective of the stage of economic development in a given country^16,17^. However, the prioritization of particular SDGs in the national context is almost foreseeable due to resource constraints that vary between countries^18^. As a result, the economic-related goals of the SDGs tend to have better progress compared to the social and environmental-related goals^5,19^, making the achievement of all 17 SDGs simultaneously remains a major challenge in many countries. Another hindrance to accomplishing the 2030 Agenda comes from the construction of the SDGs itself. The goals and targets in the SDGs framework were designed independently in silos, disregarding the potential trade-offs and synergies across and within the SDGs^20–22^. Nilsson, et al.^11^ summarized seven possible interaction types among SDGs, ranging from preventing the progress of other goals (the most negative interaction) to accelerating the achievement of other goals (the most positive interaction).

With such complex interactions, the successful implementation of the 2030 Agenda will be largely determined by how well the trade-offs and synergies across and within the SDGs are managed^22^. This necessitates an extensive analysis of SDG indicators, which unfortunately have limited data availability. The SDG Tracker Project^23^ showed that only around 38% of the SDG indicators are supplemented with global official metrics, making the rest of the SDG indicators rather difficult or even impossible to evaluate. The SDG Index^24^ emerged as an alternative approach to track the progress toward the 2030 Agenda for Sustainable Development at the global level. The SDG Index is a composite index that uses a scoring system to aggregate indicators relating to each of the 17 SDGs from a variety of publicly available sources. Despite the growing criticism of the SDG Index^25,26^, it has been widely used to analyze the interrelationship between SDGs and benchmark the national progress on SDGs^27^.

In light of the 2030 Agenda for Sustainable Development, the Inclusive Wealth (IW) Index^28^ has been proposed as a novel tool to track the progress toward sustainable development based on the accumulation of wealth involving three types of capital assets, i.e., produced, human, and natural capital. By considering those three types of capital assets, the IW Index aims to ensure that both socioeconomic development and environmental protection can be simultaneously achieved. The sustainability criterion in the IW framework will be satisfied if per capita IW increases over time, suggesting that future generations will have a greater capacity for producing goods and services that are essential for improving their well-being. Having captured the progress toward intergenerational well-being, the quantitative relationship between the IW index and the SDGs indicator remains unknown.

The notion of sustainable development requires formal evidence that intergenerational well-being can be maintained from declining over time^3^. Therefore, the SDG indicators score should not be solely used as a tool for gauging sustainability, since the progress in the goals provides no assurance that future well-being will be improved. For instance, improving the electrification rate by relying on natural gas-fired power plants combined with carbon capture storage will increase the SDG 7 score. However, such an approach will deteriorate the stock of natural capital which is unfavorable for future well-being. In this regard, the SDG Index needs to be complemented by a tool that measures changes in the wealth of nations, such as the IW Index^29^. Thus, the claim of sustainability should be characterized by positive growth in both the SDG Index score and the per capita IW Index.

Against this backdrop, there are three main objectives of this study. First, this study seeks to provide empirical evidence linking the SDGs and well-being in light of the 2030 Agenda for Sustainable Development. Instead of using subjective well-being^30^, we use the per capita IW Index as a proxy for well-being to avoid systematic economic and cognitive biases^31^. Second, efforts are made to incorporate complex interactions among the SDG Index, particularly in terms of trade-offs and synergies. Third, this study tries to understand the magnitude of each indicator area's contribution toward wealth accumulation as it relates to intergenerational well-being. Our analysis includes complex interactions between the SDG Index, especially in terms of trade-offs and synergies. Hence, this study relies on the machine learning approach, which is renowned for its remarkable capability in dealing with nonlinearity and its outstanding forecasting accuracy^32,33^. We contribute to the literature in at least two ways. First, we provide empirical evidence linking the SDG Index and the IW Index that could shed light on our current progress toward intergenerational well-being in light of the 2030 Agenda for Sustainable Development. Second, we provide a snapshot of progress in socioeconomic and environmental development where there is currently limited data so that the SDGs can be delivered in an inclusive manner.

The remainder of this paper is organized as follows: “Methods” Section describes the methodology and data, along with their limitations. “Results” Section provides the main study findings and analysis of our results. “Discussion” Section discusses our main findings and their implications for sustainability. Additionally, in this section, we attempt to connect our findings with the SDG targets. “Conclusion” Section concludes our discussion by summarizing our main findings and describing the implications of our research for society.

## Methods

*Construction of the SDGs-wealth model* To explore the global impact of SDG Index scores on well-being, we develop an SDGs-wealth model by assigning the per capita IW Index as the dependent variable and the 17 SDG Index scores as the independent variables based on the following empirical relationship:

$$IWP{C}_{it}=f(SD{G}_{it})$$

Our analysis involves complex interactions between SDG Index particularly in terms of trade-offs and synergies, hence this study relies on the machine learning approach by using the R package *xgboost*^34^. Unlike previous studies that limit the SDGs' relationship by a predetermined structural form^30,35^, our study allows unconstrained interactions between goals.

We opt to use the individual SDGs scores, instead of the SDG targets or indicators as our independent variables due to several reasons. First, the SDGs targets and indicators have limited data availability because it is not regularly collected or there is no available method to measure them. In the case of SDGs targets, the data for more than half of the 169 targets is not widely available^36^. Additionally, in the case of SDGs indicators, only 94 out of 247 SDGs indicators are widely available of which performance across the country can be directly compared^24^. Furthermore, some countries still have problems with missing data, making the SDG targets and indicators not preferable for our model. Second, the potential trade-offs between targets or indicators within the same goals are inevitable. Hence, building a model based on the SDGs targets or indicators will result in a complex model that will not be easy to comprehend. For instance, increasing agricultural productivity and income (Target 2.3) might negatively affect other targets within the same goal, such as ending all forms of malnutrition (Target 2.2) and ensuring sustainable and resilient food production systems (Target 2.4)^20^. As a result, it would be difficult for us to make a general policy recommendation regarding a certain goal due to the counteracting targets within the goal. Such complexity can be avoided by using a composite index, such as the individual SDGs scores as our independent variables. Third, we prefer to have a simple yet powerful model in terms of accuracy and interpretability. Constrained by data availability, our analysis involves a balanced panel of 147 countries from 2000 to 2019. With only 20 years of data, we prefer to have independent variables that are less than 20. Thus, compared to both the SDG targets and indicators, the 17 SDGs scores is more preferable to be used as our independent variables.

*Data sources and limitations* As a proxy of wealth, we use the IW index obtained from the Inclusive Wealth Report 2022^37^. In addition to the total wealth, we also use the disaggregated IW consisting of produced, human, and natural capital. The socioeconomic dimension of wealth is represented by produced and human capital which calculate the monetary values of man-made infrastructure and educational attainment. Additionally, the environmental dimension of wealth is represented by natural capital which calculates the monetary value of both renewable and non-renewable resources. The IW values, both the total and the disaggregated IW, are calculated in constant 2015 USD.

As a proxy of SDGs, we use the SDG Index scores obtained from the Sustainable Development Report 2022^24^. The SDG Index scores measure country’s progress in achieving the optimum SDGs performance with a maximum value of 100. We use the overall score of the SDG Index for graphical analysis, while the data of individual SDG scores are used in our SDGs-wealth model. The methodology and indicators that are used to calculate the SDG Index change every year, making us difficult to compare the scores from the current year with the previous years. To have comparable time series data, we use the backdated SDGs Index which calculated the previous year’s index by using the current year’s indicators and methods. This backdated SDGs Index is available from https://dashboards.sdgindex.org/explorer.

Our data also has some noteworthy limitations. First, although the IW index offers a comprehensive measure of wealth, it might not reflect the actual wealth of nations since there are still numerous intangible assets that cannot be monetized and excluded from the IW calculation. Similarly, the SDG Index scores might not reflect the actual performance of each indicator within an SDG, since the poor scores on some indicators can be compensated by better scores on other indicators.

*Machine learning approach* We choose the machine learning approach over other parametric and semi-parametric models because it provides several key advantages which are relevant to our research objectives. First, it can capture the nonlinear interactions between the SDGs and the per capita IW index, and second, it can identify the interaction effect between the SDGs within our model. To improve the predictive performance of our model, we rely on the boosted regression trees (BRT) method which combines both regression tree and boosting algorithms. In this method, the predictive performance of a single regression tree model is improved by growing, fitting, and combining many trees through an iterative forward stagewise process which will result in an ensemble that minimizes both bias and variance^38,39^. To obtain a robust model, particularly from overfitting, we need to simultaneously optimize three model parameters. The first parameter is the learning rate, a value between 0 and 1 which indicates how much the contribution of each tree will be reduced as it is added to the current approximation. The second parameter is the maximum tree depth, an integer that indicates the maximum depth of a tree in the model. The last parameter is the number of iterations, an integer that indicates the maximum number of boosting iterations. The optimum model was obtained at a learning rate of 0.01, a maximum tree depth of 14, and a maximum number of iterations of 5000.

Despite its outstanding predictive performance, the major shortcoming of the BRT method lies in its lack of interpretability. That is why this method is well-known as a black box model. To extract the useful information from the box and make our model explainable, we rely on the *SHapley Additive exPlanations* (SHAP) method which was developed by Lundberg and Lee^40^. The SHAP method is a model-agnostic method that explains a black box model by creating summaries of features^41^. This method is preferable since it provides both global and local interpretation of the model which allows us to grasp the average behavior of our model, through for instance the partial dependence plot, while at the same time, it enables us to explore the specific features of each instance. The SHAP method in R is available through the *SHAPforxgboost*^42^ and *shapviz*^43^ packages.

*Estimation of wealth in the Inclusive Wealth framework* In this study, we estimate inclusive wealth which comprises both marketed and non-marketed assets that provide capital stock. Adapting the sustainability concept, well-being (*V*_*t*_), is attained through the consumption of particular stock, that directly and indirectly provides goods and services for well-being. Accordingly, *K*_*(t)*_ is the productive base that comprises this capital stock, including produced capital, natural capital, and human capital, at time *t*. Natural capital accounts include forest resources (timber and non-timber), agricultural land (croplands and pastures), and fishery resources. We also consider non-renewable natural capital wealth consisting of fossil fuels (coal, natural gas, and oil), and minerals. Human capital measures include education, income, and demographic aspects such as life expectancy. Produced capital consists of a country’s infrastructure and physical assets, such as equipment, machinery, and roads. Inclusive wealth in the following equation is therefore represented by the aggregate of the production base’s *K* capital.

$${V}_{t}=V({K}_{\left(t\right),t})$$

Captured by inclusive wealth per capita, a sustainable development pathway can be achieved through non-declining intergenerational well-being, as stipulated in the following equation

$${V}_{t}={\int }_{t}^{\infty }U\left({C}_{\tau }\right){e}^{-\delta \left(\tau -t\right)}d\tau$$

Accordingly, *V*_*t*_ stands for well-being at time *t*, *U* refers to current well-being, *C*_*τ*_ represents both market and non-market consumption, and *δ* stands for the social discount rate. Therefore, future flow and capital stock are functions solely of the current capital assets. By assuming that the shadow price of each type of three main capital stocks is constant over time, we have the following equation.

$${p}_{i}(t)\equiv \frac{\partial V(t)}{\partial {K}_{i}}$$

Development is considered sustainable at $\frac{dV}{dt}\ge 0$ for all *t.* Utilizing the inclusive wealth framework, sustainability is defined in the following equation.

$$\frac{dV\left(K\left(t\right)\right)}{dt}= \sum_{i}\frac{dV\left(K\left(t\right)\right)}{\partial {K}_{i} (t)}\frac{d{K}_{i}\left(t\right)}{dt}= \sum_{i}{p}_{i}\left(t\right)\frac{d{K}_{i}\left(t\right)}{dt}\hspace{0.17em}\ge \hspace{0.17em}0$$

Accordingly, sustainable development is represented by non-decreasing inclusive wealth, if well-being converges with an economic prediction at *(t)* and is a differentiable function at ${K}_{(t)}$.

## Results

**Figure 1:**
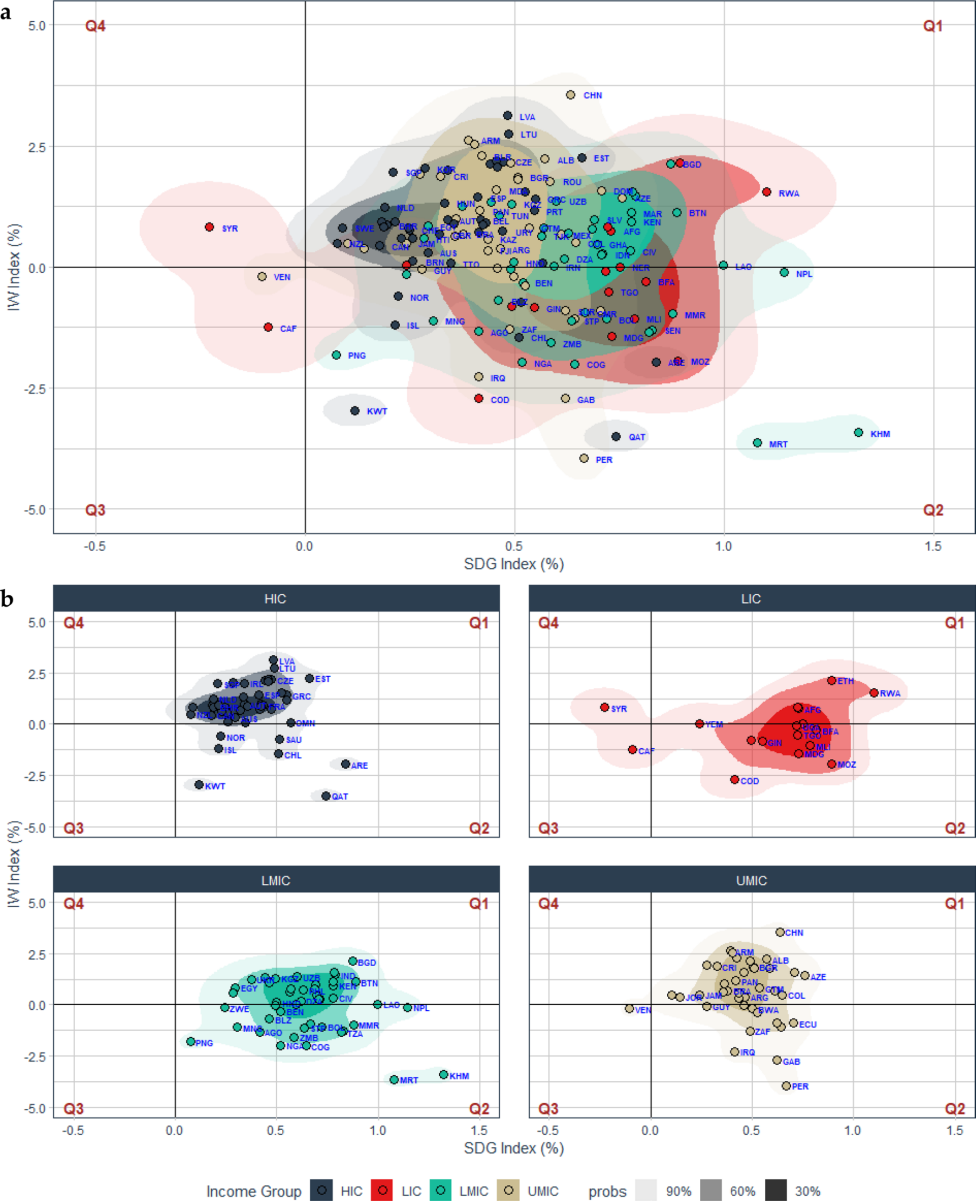
Correlation between the SDG Index and the IW Index. Visualization of annual average growth rate in SDG Index score and per capita IW Index between 2000 and 2019 using kernel density estimator (KDE). Plot generated with R package *ggdensity*^44^. Each color indicates a different country’s income group; black for high-income countries (HIC), brown for upper-middle-income countries (UMIC), green for lower-middle-income countries (LMIC), and red for low-income countries (LIC). The gradient of the colors indicates the probability based on KDE; light colors indicate a higher probability while darker colors indicate a lower probability. Q1, Q2, Q3, and Q4 represent quadrants in the plot which portray how well a particular country follows the sustainable development path. Q1 depicts the most sustainable path since the positive growth in the SDG Index score was followed by positive growth in the per capita IW Index, while Q3 represents the most unsustainable path since both the SDG Index score and the per capita IW Index were decreasing. (**a**) Progress in SDG Index score and per capita IW Index growth for 147 countries. (**b**) The progress in SDG Index score and the growth in per capita IW Index by income group.

*Linking the notion of sustainability in the SDG Index and the IW Index* Figure 1a provides an overview of countries’ progress toward sustainability over the past two decades in the framework of both the SDG Index and the IW Index. From the SDG Index’s perspective, 144 of the 147 countries assessed in this study experienced positive growth in SDG Index scores. However, only 97 out of the 147 countries in our study (65.98%) showed positive growth in both average SDG Index scores and per capita IW Index. These countries belong to quadrant 1 (Q1) which indicates good compliance with the sustainable development path since the progress in SDG indicators was complemented by formal evidence that the potential intergenerational will be increasing. Additionally, integrating the IW framework in the evaluation of sustainability will ensure that socioeconomic development can be achieved while preserving the environment. An intriguing finding was found in the case of Syria because it was the only country that experienced positive growth in the per capita IW index but a declining average score in the SDG Index, making Syria the only country that fell into quadrant 4 (Q4). Despite the increasing IW, we consider this rare case of Syria unsustainable because it has failed to efficiently allocate the accumulation of wealth to improve the well-being of the societies, particularly in terms of increasing the community’s access to nutritious food, inclusive, and equitable quality education, and affordable housing.

Without any doubt, a deviation from the sustainable development path was evident in quadrant 3 (Q3) since both the average SDG Index score and the per capita IW Index were decreasing. Two countries that fell into this category were Venezuela and the Central African Republic. However, it should be noted that in terms of absolute wealth, both Venezuela and the Central African Republic experienced positive growth in the total IW (see Fig. S1b and d in the Supplementary Information). However, their population grew at a faster rate compared to the accumulation rate of wealth. Such circumstances were regarded as underinvesting in wealth since fewer resources became available for persons. In this context, the average SDG Index showed a good agreement with the IW Index since the regress in the SDG Index scores for those two countries came from the social pillars of the SGDs which are mostly related to population growth.

Short-run sustainability is characterized by a decreasing growth in the per capita IW index, despite the positive growth in the average SDG Index score. This is portrayed in quadrant 2 (Q2) of which 47 countries belong to this category. We considered this category to have short-run sustainability because we found no formal evidence of wealth accumulation. Therefore, progress toward well-being can only be claimed for the current generations without any assurance that future generations will have at least the same capacity to maintain or improve their well-being. In other words, there is a likelihood that the current achievement of Q2 countries in terms of the SDGs will not sustain in the long-run. The significant loss in the total wealth that occurred in Q2 countries came from the underpricing of natural capital which eventually led to the overexploitation of natural resources. Moreover, those countries have failed to sufficiently compensate for the loss in natural capital through the growth in either human or produced capital (see Fig. S1 in the Supplementary Information). Accordingly, the total wealth in those nations declined, eroding future generations' productive base of the economy.

*Discrepancies of development trajectories among income groups* We carry on our analysis by dividing the countries in our study based on income groups and found different patterns of development paths between the groups (see Fig. 1b). The majority of high-income countries (HIC) (39 out of 46 countries) belong to Q1, indicating good compliance with the sustainable development path evaluated from both the SDG Index and the IW Index framework. A rather similar pattern was found for upper-middle-income countries (UMIC) of which 27 out of 39 countries fell into Q1. However, as portrayed in Fig. 1b, the probability of a particular country falling into Q1 decreases along with decreasing income levels. For the case of lower-middle-income countries (LMIC), 26 out of 45 countries belong to Q1 while for low-income countries (LIC), only 5 out of 17 countries were found in Q1. These findings justify that the economy's scale effect, which usually appears in the early stage of economic development, tends to be harmful to sustainability because it is characterized by massive extraction of natural resources that exceed their regeneration rates. However, as the economy grows, the composition and technological effects become available, transforming the economy from a resource-intensive economy to a technology-intensive economy^45^. Failure to do so will make environmental degradation inescapable, which is unfavorable for sustainable well-being.

**Figure 2:**
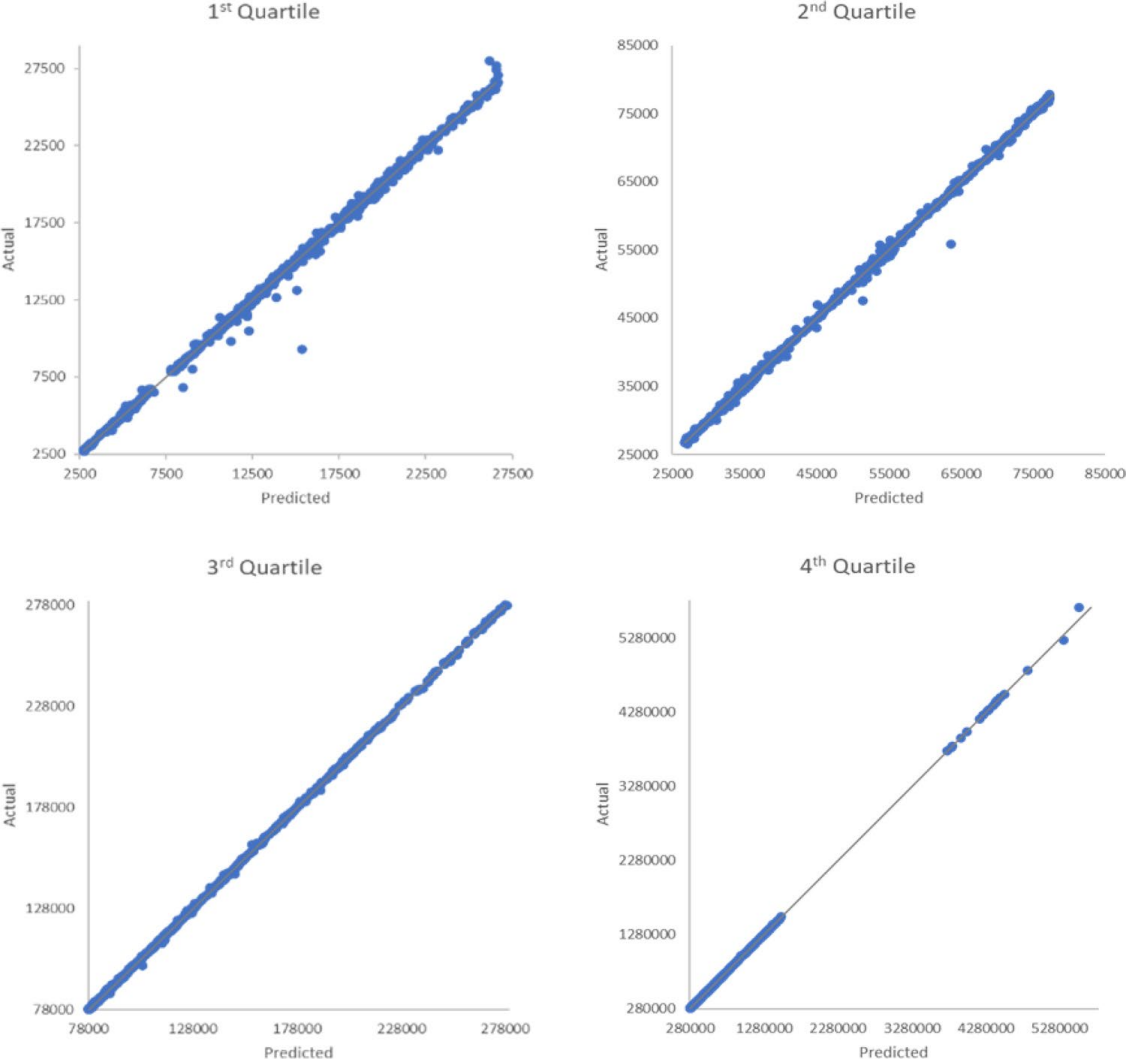
Predictive performance of the SDGs-wealth model. The SDGs-wealth model was analyzed using the R package *xgboost*^34^. The evaluation of the model performance is divided by quartile. The y-axis represents the actual value, while the x-axis represents the predicted value. The grey line represents the regressed diagonal line. The model’s predictive performance is determined by how far the points deviate from the diagonal line. A good fit is indicated by the points that are close to the diagonal line.

*Establishing the link between the SDG Index and the IW Index through the SDGs-wealth model* By employing the machine learning method, we aim to establish the link between the SDGs and the IW index through the SDGs-wealth model. We found that the accumulation of wealth, which is proxied by the per capita IW Index, was well predicted by the changes in SDG Index scores. Figure 2 shows that our model demonstrates excellent predictive performance for all ranges of quartiles, with the mean absolute error and mean absolute percentage error of 11.41 and 2.84 × 10^−4^, respectively.

**Figure 3:**
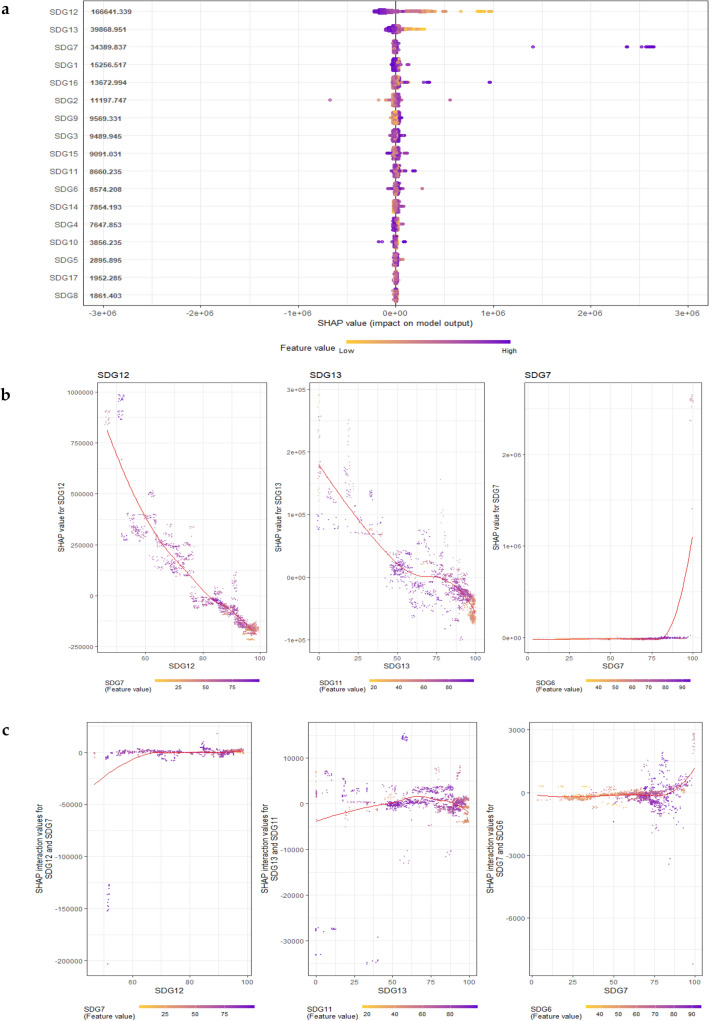
Global interpretation of the SDGs-wealth model based on the SHAP values. The Shapley additive explanations (SHAP) values and the plot were generated with the R package *SHAPforxgboost*^42^. The color represents the SDG Index score from low to high (yellow for a low score, purple for a high score). (**a**) The SHAP summary plot. The y-axis represents the Goals, while the x-axis represents the SHAP value. The dots in the summary plot represent SHAP values that correspond to particular goals. The goals are ordered according to their influence on prediction. (**b**) The SHAP dependence plot. The x-axis represents the SDG Index score, while the y-axis represents the SHAP value for individual goals. The red line represents the locally estimated scatterplot smoothing (LOESS) curve. (**c**) The SHAP interaction plot. The x-axis represents the SDG Index score, while the y-axis represents the SHAP interaction value between two selected goals which was chosen based on the highest conditional variances. The red line represents the locally estimated scatterplot smoothing (LOESS) curve.

We also identified discrepancies between the SDGs in accumulating the per capita IW Index. From Fig. 3a, we can see that the top three predictors of the per capita IW Index in our model were environmental-related SDGs which include Goal 12 (Responsible Consumption and Production), Goal 13 (Climate Action), and Goal 7 (Affordable and Clean Energy). These were followed by socioeconomic-related SDGs i.e., Goal 1 (No Poverty), Goal 16 (Peace, Justice and Strong Institutions), and Goal 2 (Zero Hunger). Other SDGs also contributed to the predictive performance of our model but with less magnitude, with the bottom predictor being Goal 8 (Decent Work and Economic Growth).

In addition to the rank of importance, the SHAP summary plot also provides the direction of influence from each SDG Index score. Figure 3a shows that the specific impacts of Goal 12 and Goal 13 on the average prediction of the model were surprisingly negative, suggesting that countries with high Goal 12 and Goal 13 scores were associated with low per capita IW Index. These results were confirmed further by the SHAP dependence plots which are provided in Fig. 3b. Our findings are in a good agreement with the previous studies of Lusseau and Mancini^46^ who found the negative impacts of Goal 12 and Goal 13 either on a particular SDG or on the SDGs as a whole, by using the sustainomes network of interactions for SDGs. Similar results were also reported by De Neve and Sachs^30^ who found the negative impacts of Goal 12 and Goal 13 on subjective well-being. These unexpected results came from the fact that most of the developed economies performed better than the developing economies in terms of wealth accumulation. However, most of the indicators that were used to calculate Goal 12 and Goal 13 scores were based on the waste footprint of the economy, such as the amount of municipal solid waste, plastic waste, and greenhouse gas emissions. As a result, the developed economies tend to have lower average Goal 12 and Goal 13 scores than the developing economies since they extract more resources to fuel their economies and produce more waste as byproducts of their economic activities^47,48^. Those paradoxical scores have been the subject of criticism in the previous study regarding the appropriateness of the SDG Index scoring^25^.

In contrast to Goal 12 and Goal 13, we found a positive correlation between Goal 7 and the per capita IW Index. In line with the existing literature on energy and sustainability^49–51^, our finding highlights the significant role of energy justice and renewable energy in promoting sustainable well-being. Similarly, the impact of Goal 2 on wealth accumulation was positive, refuting the view that the development of the agricultural sector to combat zero hunger threatens the sustainability of the environment^20^. This finding was not beyond expectation, since the IW framework takes into account the agricultural sector as part of the natural capital that contributes to the total wealth^28^. Hence, investing more in agricultural land does not necessarily harmful to sustainability, as long as the loss in the social value of other types of natural capital can be compensated by the gain in the social value from the agricultural sector.

We could not establish a well-defined relationship between the SDG Index score and the per capita IW Index for the rest of the goals by simply relying on the SHAP summary plot. Instead, we also need to consider the SHAP dependence plot as provided in Fig. S2. Based on the SHAP dependence plot, we found that the individual impact of Goal 17 (Partnerships for the Goals) on the per capita IW Index was positive. However, a negative correlation was found for the case of Goal 10 (Reduced Inequalities). A nonlinear interaction that follows a U-shaped curve was found for the case of Goal 16, Goal 9 (Industry, Innovation, and Infrastructure), Goal 3 (Good Health and Well-Being), and Goal 11 (Sustainable Cities and Communities). An opposite relationship was found for the case of Goal 1, Goal 14 (Life Below Water), and Goal 15 (Life on Land) in which the relationship between the SDG Index score and the per capita IW Index follows an inverted U-shaped curve. Furthermore, a more complex relationship was found for the case of Goal 4 (Quality Education), Goal 5 (Gender Equality), and Goal 6 (Clean Water and Sanitation), which follows the N-shaped curve, and Goal 8 (Decent Work and Economic Growth) which follows the inverted N-shaped curve.

*Interactions between the SDGs within the SDGs-wealth model* The aforementioned discussion regarding the features of the SDGs-wealth model was based on the individual impacts of each goal on per capita IW, disregarding any possible interaction between the goals. To analyze the possible interactions between the goals in our SDGs-wealth model, we rely on the SHAP interaction plot which portrays the difference between the SHAP values for a particular goal in the presence and/or absence of other goals (Fig. 3b). We are going to focus on the top three predictors of our model based on the SHAP summary plot, i.e., Goal 12, Goal 13, and Goal 7, since the SHAP feature importance values for other goals were rather small. Figure 3b shows that Goal 12 had the most substantial interaction with Goal 7, while Goal 13 and Goal 7 were found to have substantial interactions with Goal 11 and Goal 6, respectively. Based on the LOESS curve, the combination effect between Goal 12 and Goal 7 on per capita IW took the form of a logarithmic growth curve. This finding implies that the per capita IW increased rapidly at the low values of Goal 12, but then the positive impact would diminish along with the increasing values of Goal 12. In contrast, the joint effects between Goal 7 and Goal 6 took the form of an exponential growth curve, suggesting that the per capita IW grew slowly at the low values of Goal 7, but then it accelerated massively with the increasing values of Goal 7. For the case of Goal 13, we found an inverted U-shaped relationship suggesting that the positive impact of the joint effects between Goal 13 and Goal 11 occurred only temporarily. Once it reached its peak, an increase in Goal 13 would lead to a decrease in per capita IW, particularly for the low values of Goal 11.

We carry on our analysis further to scrutinize different types of goals interactions within our model. For this purpose, we make a comparison between the trend of the LOESS curve in the SHAP interaction plot (Fig. 3c), which represents the joint effects between two goals, and the trend of the LOESS curve in the SHAP dependence plot (Fig. 3b), which represents the individual effect of a particular goal. If there is a noticeable difference in the trend of the LOESS curve between the two plots, then we can argue that either a trade-off or synergy exists between the two goals, otherwise, the interaction is indeterminate. For the case of Goal 12, we found that the downtrend of the LOESS curve on the SHAP dependence plot was reversed in the presence of Goal 7, suggesting a synergy between Goal 12 and Goal 7 in accumulating wealth. Synergy was also found in the case of Goal 13 and Goal 11, although at the high values of Goal 13 the interaction became indeterminate. For the case of Goal 7, the joint effects with Goal 6 were also indeterminate, since there was no significant change in the trend of the LOESS curve between the SHAP dependence plot and the SHAP interaction plot*.*

**Figure 4:**
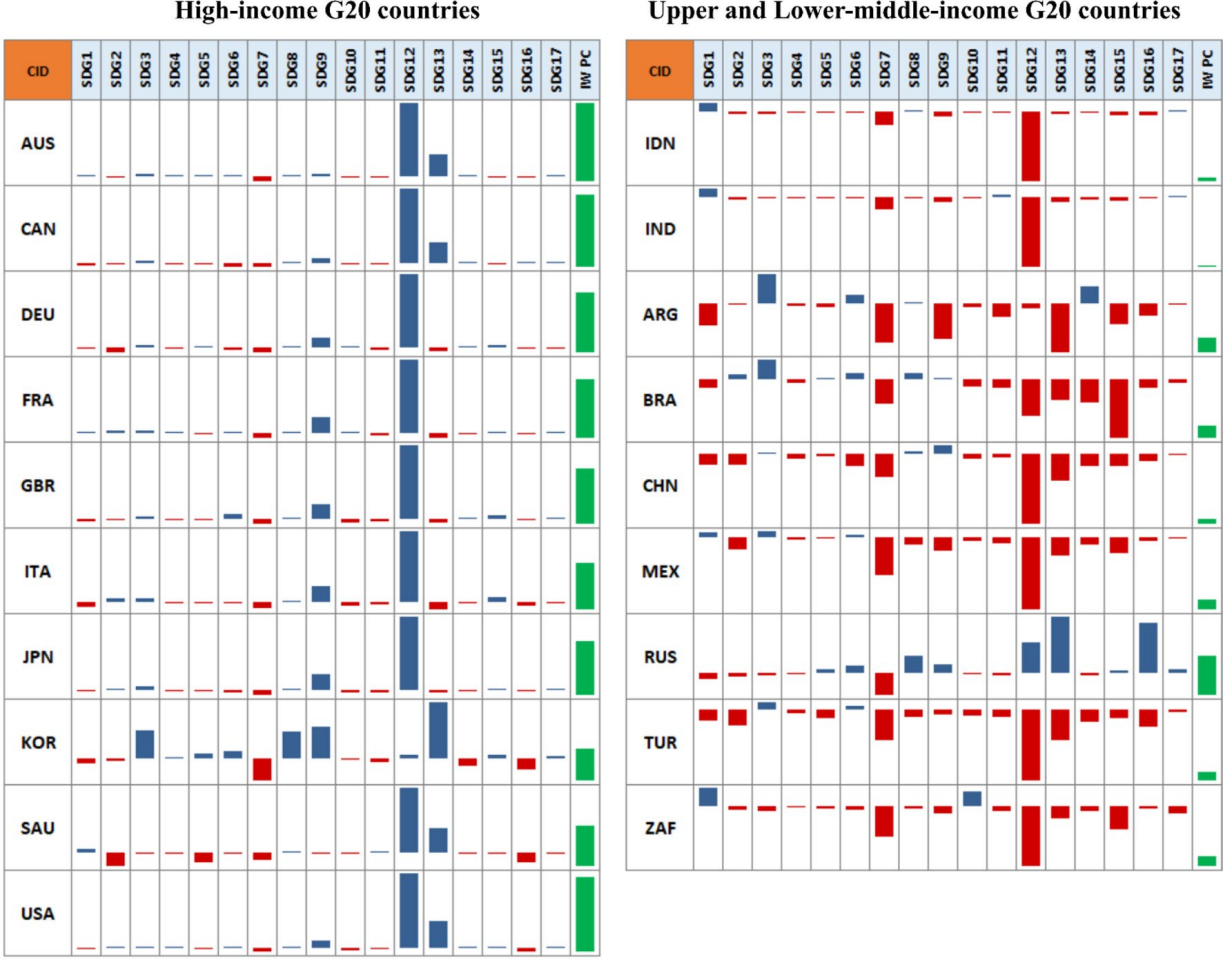
Local interpretation of the SDGs-wealth model based on the SHAP values for G20 countries. Decomposition of a single prediction in our SDGs-wealth model using the SHAP force plot provided by the R package *shapviz*^43^. The SHAP values from the SHAP force plots were extracted and modified into column sparklines. The blue bar indicates a positive contribution to the predicted value, while the red bar indicates a negative contribution. The bar's length indicates the contribution to the predicted value relative to other independent variables. The green bar indicates the predicted value (per capita IW Index).

*Wealth accumulation patterns at the national level* Shifting from the global analysis, we examine different patterns of wealth accumulation at the national level. For this purpose, we employ the SHAP force plot, a local model-agnostic method, which decomposed the predicted value of the per capita IW into contributions from each goal. To conserve space, we modified the SHAP force plot into column sparklines, and limit the scope of countries in Fig. 4 only to G20 countries. The modified SHAP force plots for other countries are provided in Supplementary Information 3.

From Fig. 4, we can see that Goal 12 was the most influential predictor of the per capita IW, with the exceptions of Russia, Korea, Argentina, and Brazil where Goal 13 and Goal 15 were found as the most influential predictors. We also found discrepancies in wealth accumulation patterns between high and middle-income countries. In the case of high-income countries, the impact of Goal 12 on wealth accumulation was positive. However, a negative impact of Goal 12 on wealth accumulation was found for upper and lower-middle-income groups, except for Russia. As regards the global panel (Supplementary Information 3), we found some similarities with the case of G20 countries. In the vast majority of high-income countries, the impacts of Goal 12 on wealth accumulation were positive. Whereas in the middle and low-income groups, Goal 12 was negatively associated with wealth accumulation. Contradicting patterns between income groups were also evident in Goal 13, although they were not as obvious as in Goal 12. The majority of high-income countries such as Australia, Canada, South Korea, Saudi Arabia, and the USA, had a relatively low SDG Index 13 score, yet the contribution of this goal to wealth was positive. Interestingly, the impacts of Goal 7 on wealth accumulation in G20 countries were negative for all income groups. For the global panel, only 5 out of the 147 countries in our study showed positive correlations between Goal 7 and wealth accumulation. In line with the previous study^50^, these findings suggest that the dilemma between energy and climate objectives remains a major development issue worldwide.

## Discussion

Global efforts to establish a more sustainable development trajectory under the 2030 Agenda for Sustainable Development have been elusive, even after seven years of the implementation of the SDGs. This has raised the question regarding the sustainability of the SDGs themselves. Although we acknowledge the shortcoming of the SDGs, our study does not dismiss nor criticize the notion of sustainability in the SDGs framework. Instead, we establish the link between the SDGs and the IW Index to ensure that the SDGs are indeed sustainable both in the short and long-run.

The contributions of the IW framework are twofold. Firstly, the IW Index ensures the integrated and indivisible nature of the SDGs by providing a framework that balances the progress in each SDG, particularly between investment in capital goods and enabling assets. Additionally, the IW framework opposes development policies focusing solely on economic growth in terms of GDP per capita, since it was least likely to accumulate wealth. As a result, both socioeconomic development and environmental sustainability can be simultaneously achieved without posing the dilemma of choosing between the two. Recent studies that track the progress of world nations towards all 17 SDGs reported that socioeconomic-related SDGs were progressing better than the environmental-related SDGs^5,19^. However, our findings show that environmental-related SDGs have a greater influence on wealth accumulation compared to socioeconomic-related SDGs. Hence within the IW framework, the nations will be navigated to accelerate the progress of environmental-related SDGs for creating a balance with the socioeconomic-related SDGs. By doing so, the wealth of the nations will be benefited in at least three ways: first, the economy will be dematerialized, second, the negative externalities from economic activities that erode the total wealth will be avoided, and third, the rate of capital accumulation will increase.

Secondly, the IW framework accommodates the inevitable trade-offs and synergies between the SDGs. Sustainability assessment in the IW framework is not based on the individual achievement of each SDG but it is based on the accumulation of total wealth in terms of the per capita IW Index. Our findings show that despite their unsustainable consumption and production patterns, most high-income countries have managed to compensate for the significant social loss from environmental degradation (low environmental-related SDGs score), with sufficient investment in either produced or human capital (high socioeconomic-related SDGs score). As a result, their total wealth can be maintained from declining, which is in concordance with the weak sustainability criteria in the IW framework. Unfortunately, the majority of the countries that belong to middle and low-income groups have failed to make such compensation, resulting in a noticeable decline in their total wealth. Our findings also show that trade-offs and synergies between the SDGs transpire in a rather complex manner. The net effects of the interactions on total wealth were also varied and could not be simply depicted by a linear relationship. Hence, under the IW framework, instead of focusing on how the SDGs interact with each other, we only need to consider the net effect of the interactions on total wealth.

However, these findings are not without caution, since the social loss from environmental degradation in the high-income countries was compensated less and less each year by net increases in either produced or human capital^28^. If this trend continues, then the sustainable development trajectories in high-income countries will not sustain in the long-run. In this regard, the IW framework will navigate the developed and developing economies in two different ways. First, the developed economies are once again urged to accelerate the progress in environmental-related SDGs, e.g., by increasing resource efficiency and shifting to renewable resources since natural resources are finite and becoming scarce. Second, the developing economies are urged to make more investments in produced and human capital to accelerate the progress in socioeconomic-related SDGs.

We carry on our discussion further to touch on the SDGs’ targets that are relevant to our main findings. We will limit our discussion to the targets that are directly related to the calculation of SDGs Index score in our SDGs-wealth model. To begin with, the positive impact of Goal 7 on wealth creation cannot be separated from the increasing share of renewable energy in the global energy mix, which is depicted by Target 7.2. In the IW framework, increasing the share of renewable energy will be beneficial to wealth by at least twofold^52^. Firstly, similar to non-renewable, renewable energy infrastructures will be considered as produced capital. Hence, making new investments in renewable energy infrastructure will positively influence total wealth through produced capital. Secondly, unlike non-renewable energy, input for renewable energy infrastructures comes from renewable resources that will not deplete natural capital. However, shifting to renewable energy might hinder the progress of Target 7.1 to provide universal access to affordable energy since renewable energy tends to have higher capital costs compared to fossil fuels^53,54^.

Shifting to Goal 12, the most relevant target that represents the SDG Index score for Goal 12 is Target 12.5: substantially reduce waste generation through prevention, reduction, recycling, and reuse. As a result, the impact of Goal 12 on wealth creation was found to be negative since rich countries tend to produce more waste (lower Goal 12 score) and at the same time accumulate more wealth (higher IW index). However, despite the importance of Target 12.2: sustainable management and efficient use of natural resources on wealth creation and well-being, it has not been explicitly included in the calculation of the Goal 12 score. Natural capital is an essential component of wealth, and the achievement of the SDGs, either directly or indirectly, will depend largely on the sustainable stock of natural capital^55,56^. Hence, future work on the SDG Index scores needs to take into account indicators that measure the changes in natural capital. By doing so, we expect to see different impacts of Goal 12 on wealth creation in our SDGs-wealth model. A rather similar case was found in Goal 13, where the calculation of the score was based mainly on carbon dioxide emissions-related indicators. As a result, rich countries that are likely to emit more carbon dioxide emissions tend to have a lower Goal 13 score. Integrating climate change measures into national policies, strategies, and planning (Target 13.2) is expected to decouple economic growth from carbon dioxide emissions. However, it should be done with caution so that the utility of current and future generations can be maintained from being declined^50^.

## Conclusion

Despite its promising concept for sustainability, the SDGs framework has drawn widespread criticism. Our study aimed to establish a link between the SDGs and the IW index in order to overcome the SDGs' shortcomings and ensure that the SDGs can be delivered in an inclusive manner. We developed an SDGs-wealth model involving panel data on the IW index and SDGs Index of 147 countries for 2000–2019. Utilizing the machine learning approach, we found that the changes in wealth were well predicted by the changes in the individual SDG scores. The three main predictors of wealth were Goal 12 (responsible consumption and production), Goal 13 (climate actions), and Goal 7 (affordable and clean energy). As expected, we found that promoting access to affordable, reliable, sustainable and modern energy was beneficial for wealth accumulation. However, implementing the sustainable consumption and production patterns and combating climate change were found to be unfavorable for wealth creation. Out of 147 countries in our study, 50 countries were found to deviate from the sustainable development trajectories either in the short or long run.

*Implications for researchers* The vast majority of the existing literature on SDGs concentrates on analyzing the trade-offs and synergies between and within the SDG indicators and how to formulate policies that optimize the synergies while resolving the trade-offs between the goals. Our SDGs-wealth model shows that the changes in wealth are well predicted by the changes in the SDG Index score. Supported by this finding, in the pursuit of the 2030 Agenda, we provide new insight for researchers and encourage them to put greater efforts into measuring the changes in wealth, instead of focusing on the complex interactions between the goals. Our study also encourages researchers to develop a more comprehensive tool for measuring the progress of economic development that is applicable both at the national and global levels. To date, the IW index might be considered as one of the most reliable measures of sustainable development, however, it is not without flaws.

*Implications for policymakers* Successful implementation of the 2030 Agenda will largely depend on how the nation’s progress toward sustainable development paths is monitored and evaluated. This will inform the policymakers regarding where the nations are currently standing, what kind of challenges the nations have to deal with in achieving the goals, and enable the policymakers to determine which particular policies offer the greatest potential for achieving all of the goals. Our study presents the IW framework, which offers a more comprehensive assessment of sustainability based on the accumulation of capital assets that matter for well-being. We suggest policymakers to move beyond the GDP and start to adopt the IW index in the policy-making process. Coupled with the SDG framework, the IW index will complement the shortcomings of the SDGs and safeguard the inclusive development of the three main pillars, i.e., economy, society, and environment, which in turn will lead to the sustainability of economic development both in the short and long-run.

*Future works* Our study is greatly constrained by the availability of data. With the current dataset, we are unable to carry out a deeper and more comprehensive analysis at the targets and indicators level. Along with the increasing time span and coverage of the SDGs dataset in the future, a similar SDGs-wealth model can be applied while putting greater focus on assessing the impacts of each individual SDG, in conjunction with its related targets and indicators, on wealth creation. With regard to the methodology, our analysis relies on the nonparametric machine learning approach. Therefore, although we have managed to identify the correlations and interactions between variables and distinguish which variables act as the most significant predictors of wealth, we cannot make any inferences regarding the causality between variables. In this regard, future research needs to consider using the parametric panel data approach to get a robust statistical inference that can verify and complement the results from the nonparametric approach. Furthermore, regarding the dependent variable, future research might consider to use other proxies for well-being in the SDGs-wealth model.

## Supplementary Information

Supplementary Information.

## Data Availability

The Inclusive Wealth data obtained from the Inclusive Wealth Report 2022 is not freely available, however, the datasets used and/or analyzed during the current study may be made available from the corresponding author upon reasonable request. The SDG Index scores obtained from the Sustainable Development Report 2022 are freely available and can be downloaded from https://dashboards.sdgindex.org/explorer.
