# Author Correction: Assessing the United Nations sustainable development goals from the inclusive wealth perspective

**DOI:** 10.1038/s41598-023-29318-0

**Published:** 2023-02-03

**Authors:** Yogi Sugiawan, Robi Kurniawan, Shunsuke Managi

**Affiliations:** 1 National Research and Innovation Agency of Indonesia (BRIN), Gedung 90 , Kawasan Puspiptek Serpong, South Tangerang, 15340 Indonesia; 2 Ministry of Energy and Mineral Resources, Jalan Pegangsaan Timur No. 1a, Jakarta, 10320 Indonesia; 3 Urban Institute, Kyushu University, 744 Motooka, Nishi‑ku, Fukuoka, Japan

Correction to: *Scientific Reports*, 10.1038/s41598-023-28540-0, published online 28 January 2023

The original version of this Article contained an error in Reference 37,

37. Barbier, E. B. *et al.* Inclusive Wealth Report 2022 Executive Summary. (United Nations Environment Programme, 2022).

now reads:

37*.* UNEP. Inclusive Wealth Report 2022 Executive Summary. (United Nations Environment Programme, 2022).

The original Article has been corrected.

