# Supplementary material for: Assessing the United Nations sustainable development goals from the inclusive wealth perspective
Source: Sci Rep. 2023 Jan 28;13:1601. doi: 10.1038/s41598-023-28540-0 (PMC9884189; doi:10.1038/s41598-023-28540-0)
Supplement: Supplementary file 1 — Supplementary Information. [file 41598_2023_28540_MOESM1_ESM.docx]

Supplementary Information for

**Assessing the United Nations Sustainable Development Goals from the Inclusive Wealth perspective**

Yogi Sugiawan ^a^, Robi Kurniawan ^b^, Shunsuke Managi ^c*^

^a^ National Research and Innovation Agency of Indonesia (BRIN), Jl. Kuningan Barat, Mampang Prapatan, Jakarta 12710, Indonesia

^b^ Ministry of Energy and Mineral Resources, Jalan Pegangsaan Timur No. 1a, Jakarta 10320, Indonesia

^c^ Urban Institute, Kyushu University, 744 Motooka, Nishi-ku, Fukuoka 819-0395, Japan

^*^ Corresponding author email: managi.s@gmail.com

a

b

c

d

**Fig. S1 Measuring the changes of the total, per capita, and disaggregated per capita wealth: average annual growth rates from 2000–2019.** The sparklines portray the annual growth of wealth during the period 2000-2019 and the year 2000 is set as a base year. The upward green triangle indicates positive growth, while the downward red triangle indicates negative growth. **a.** High-income countries. **b.** Upper-middle income countries. **c**. Lower-middle income countries. **d**. Low-income countries


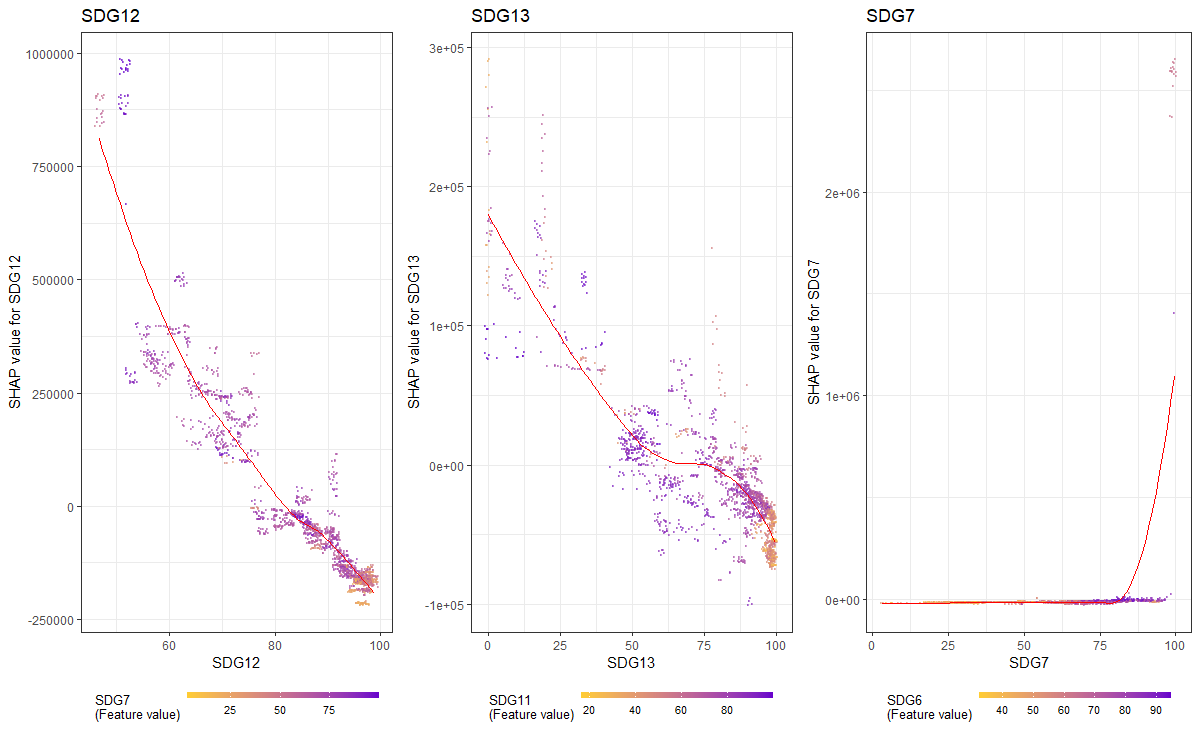

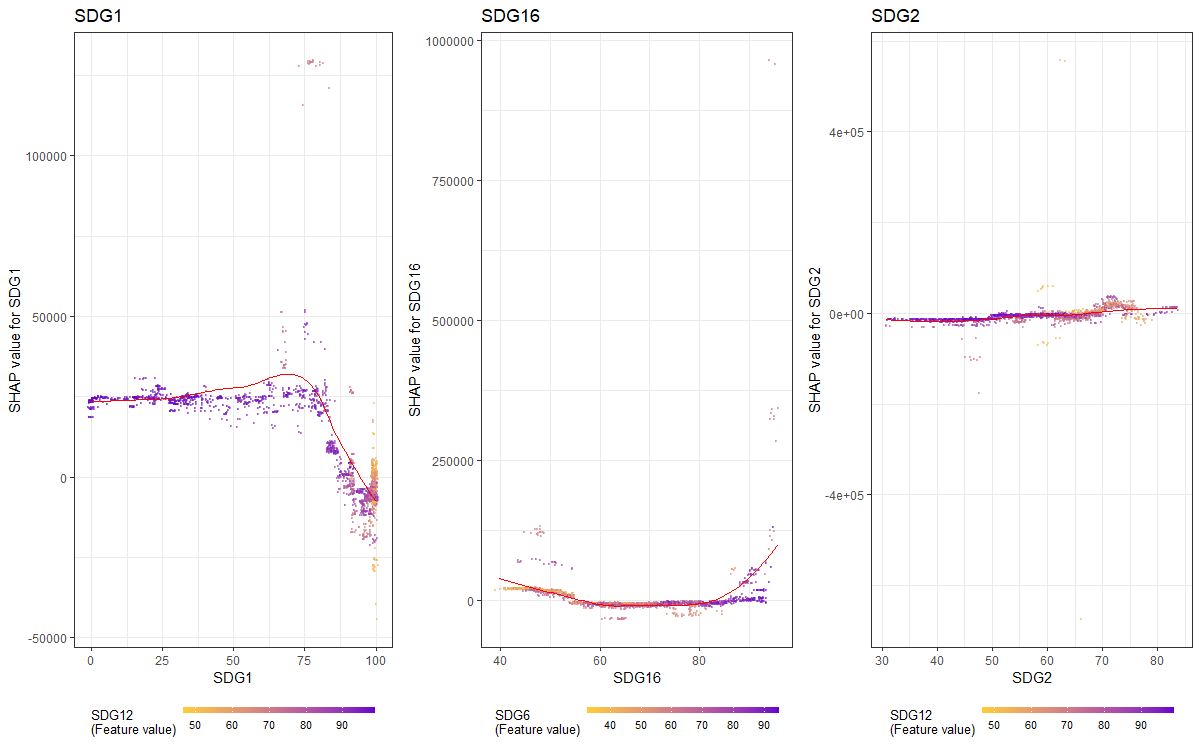


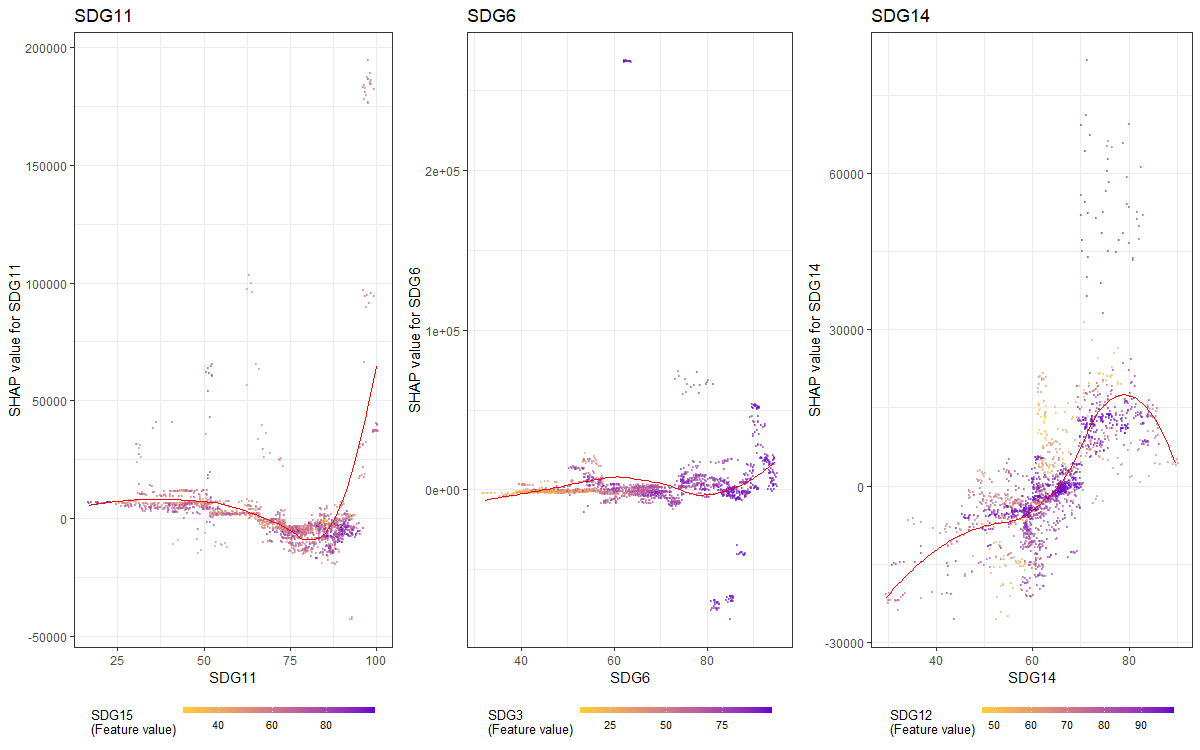

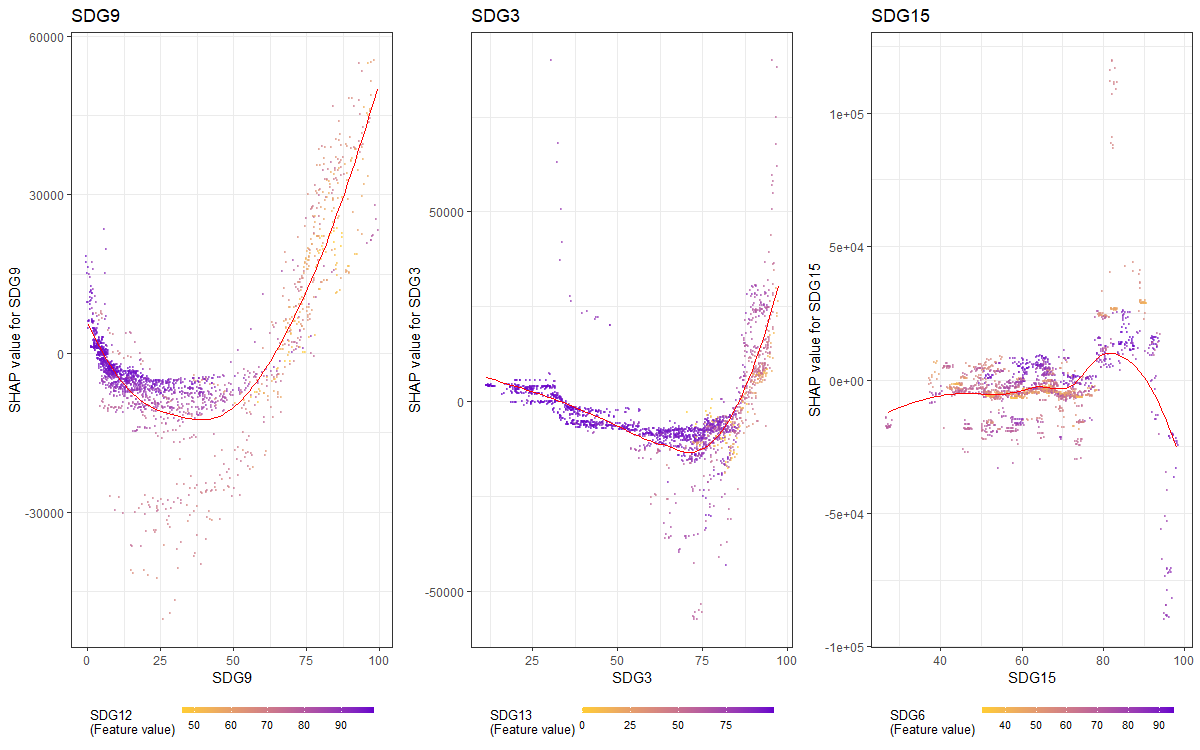


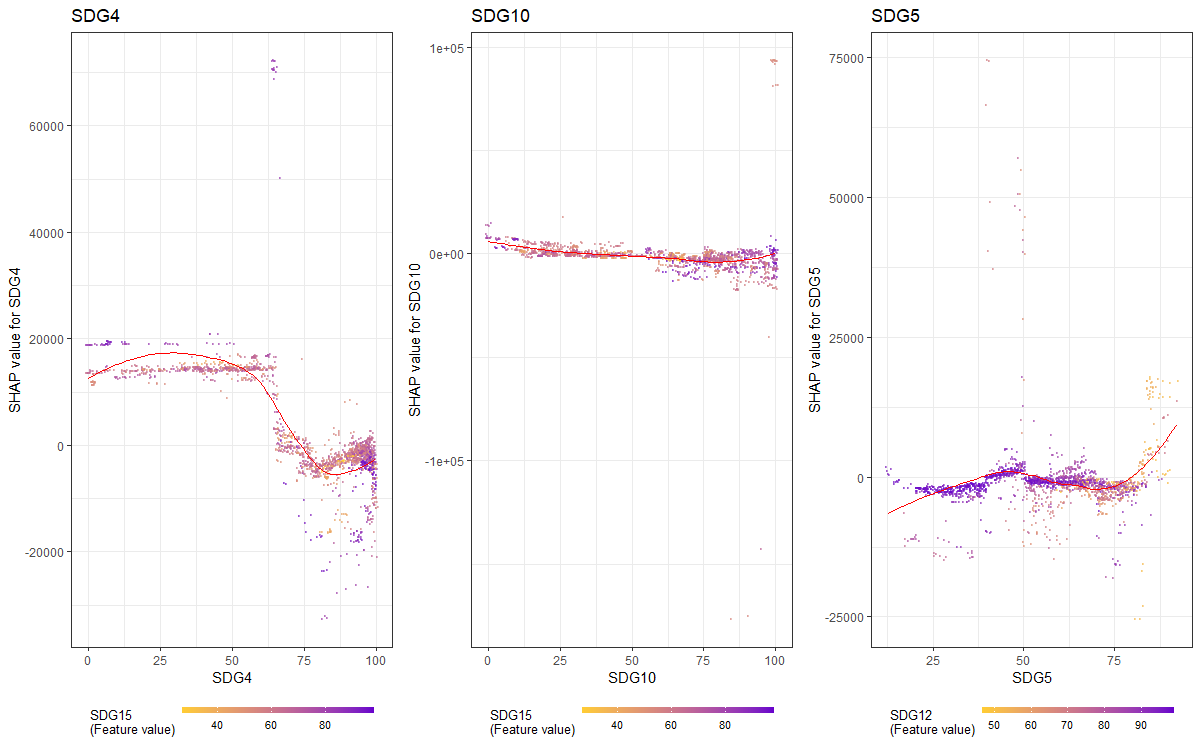


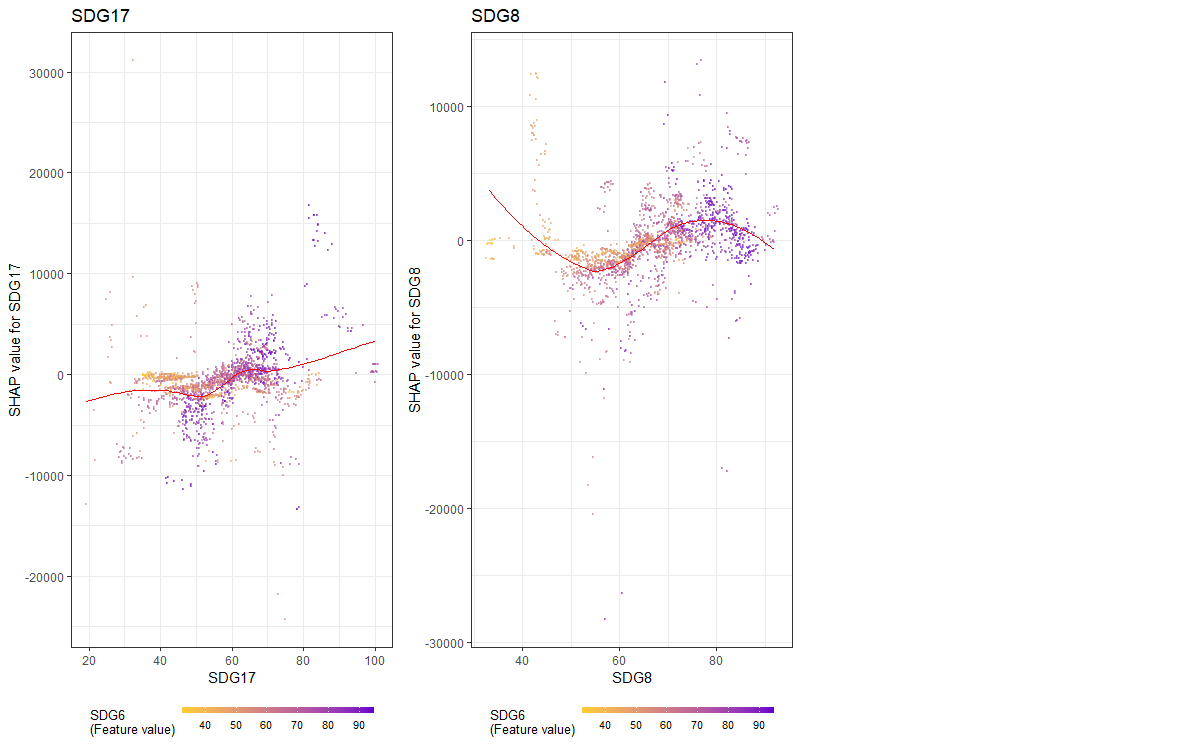


**Fig. S2 Global interpretation of the SDGs-wealth model based on the SHAP dependence plot.** The color represents the SDG Index score from low to high (yellow for low score, purple for high score). The x-axis represents the SDG Index score, while the y-axis represents the SHAP value for individual goals. The red line represents the locally estimated scatterplot smoothing (LOESS) curve.

a


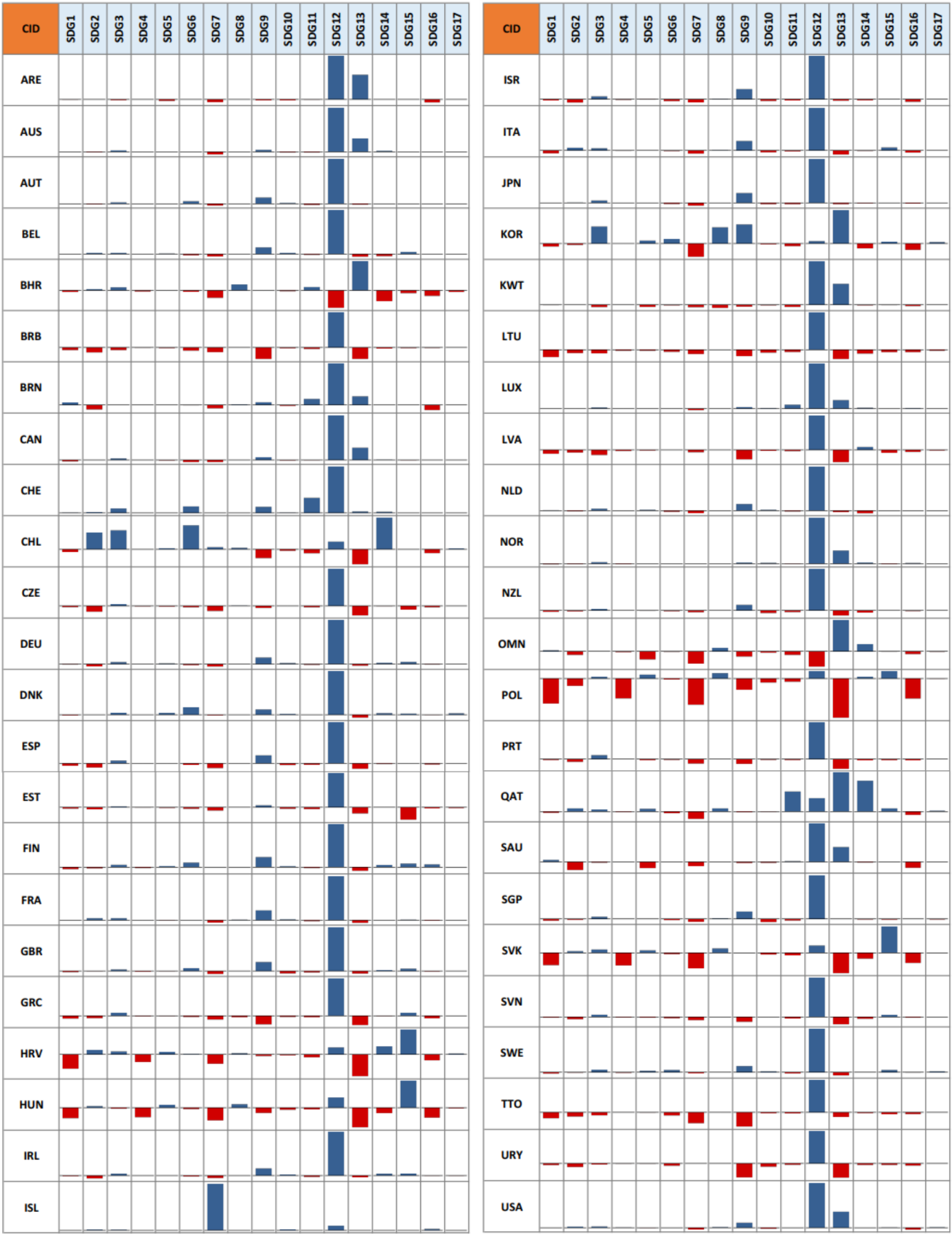


b


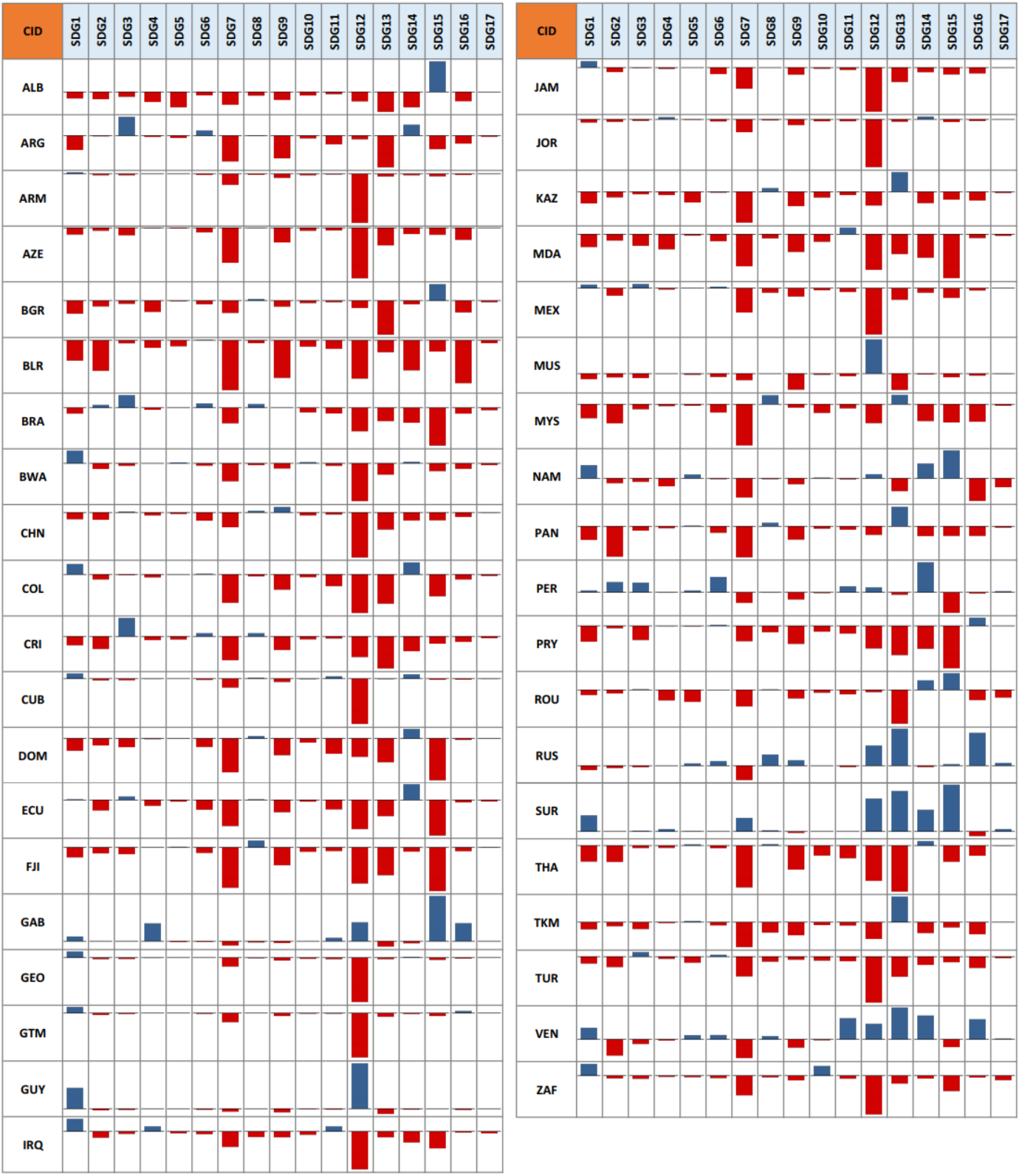


c


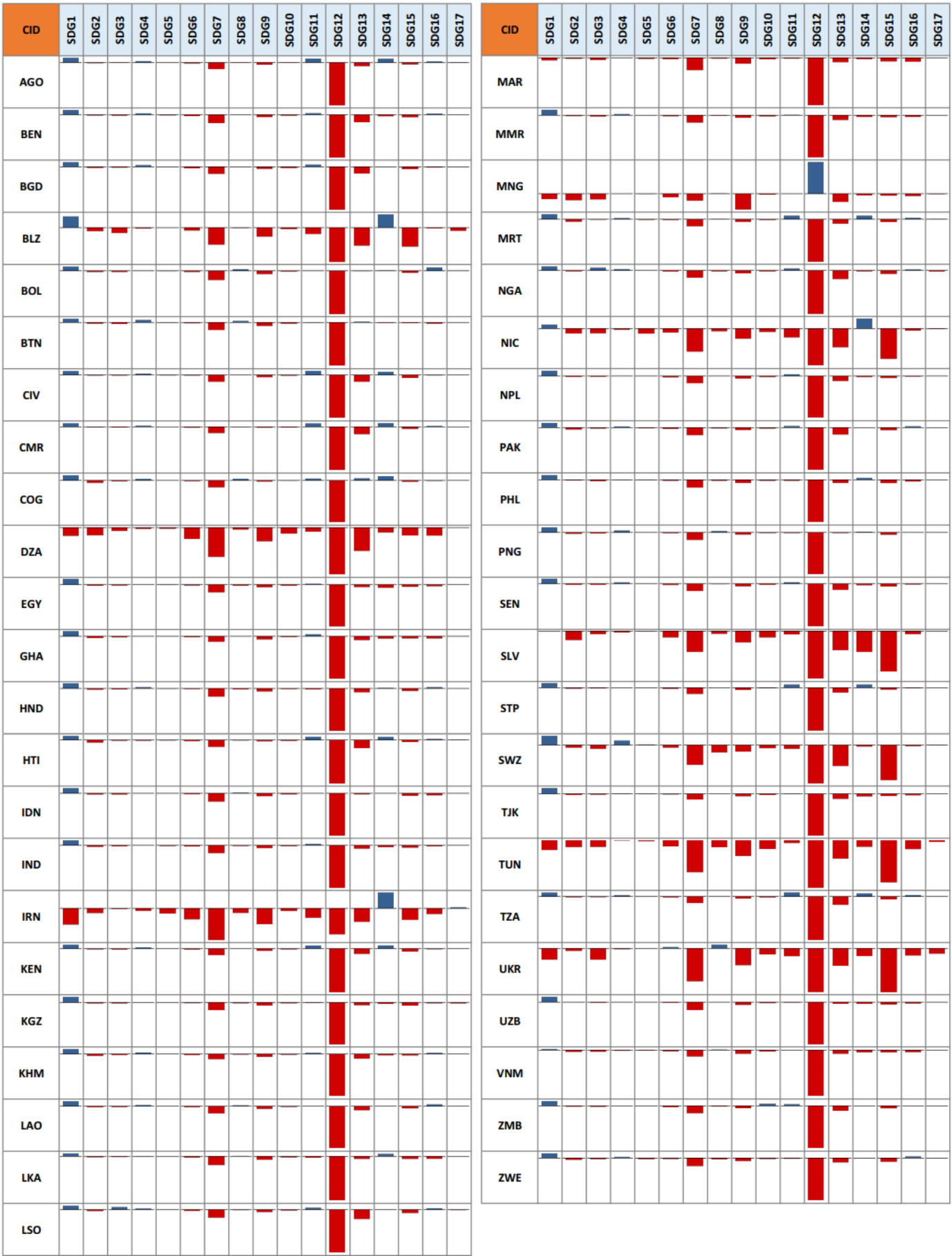


d


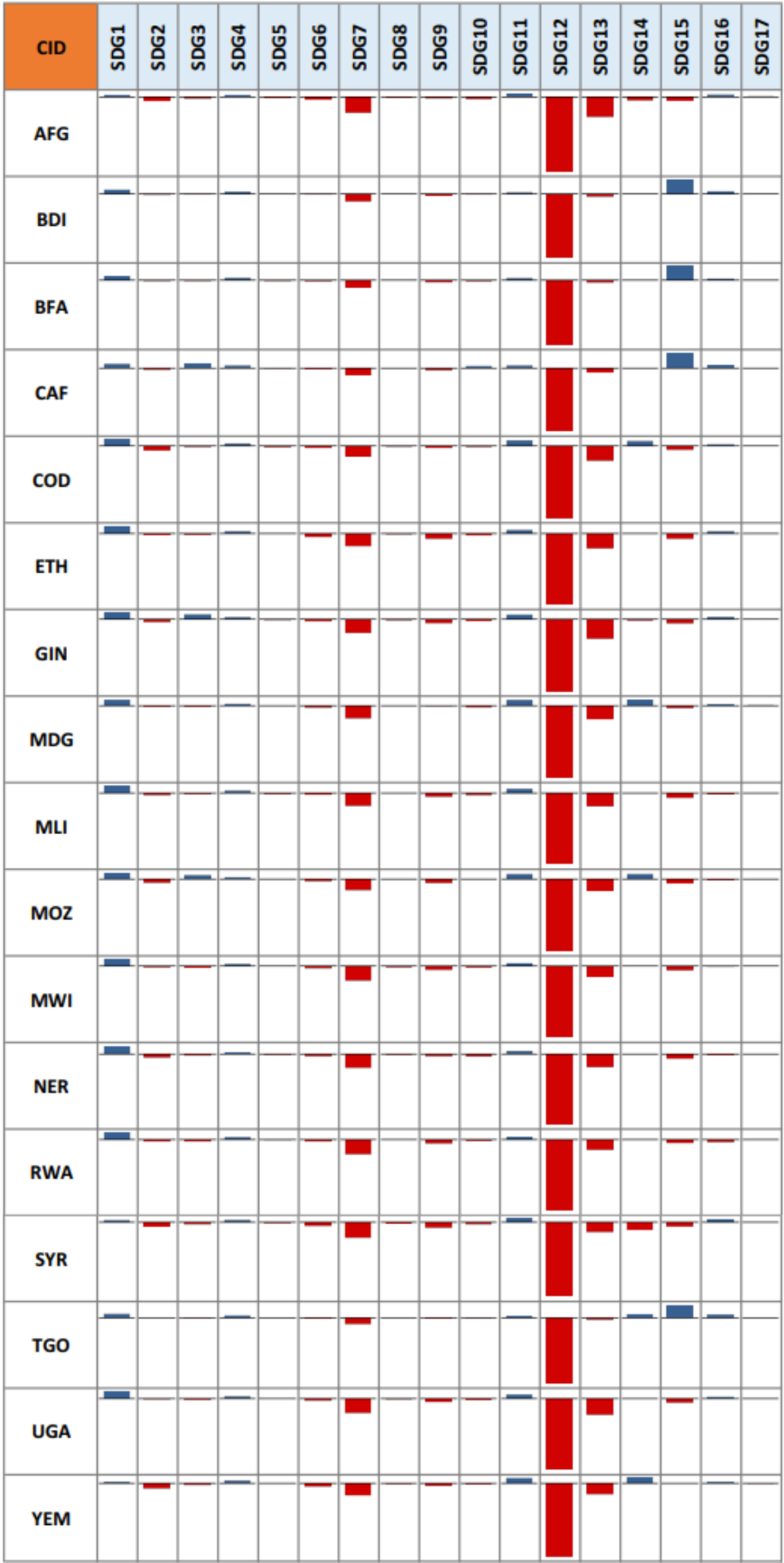


**Fig. S3 Local interpretation of the SDGs-wealth model based on the SHAP values.** The SHAP values from the SHAP force plots were extracted and modified into column sparklines. The blue bar indicates a positive contribution to the predicted value, while the red bar indicates a negative contribution. The bar's length indicates the contribution to the predicted value relative to other independent variables. The green bar indicates the predicted value (per capita IW Index). **a.** High-income countries. **b.** Upper-middle income countries. **c**. Lower-middle income countries. **d**. Low-income countries
